# Botanical Report

**Published:** 1813-03

**Authors:** 


### BOTANICAL REPORT.

HAVING been prevented, by more urgent contributions, from continuing this Report regularly, we have suffered considerable arrears to accumulate upon our hands; a debt which we shall endeavor now at least to begin to discharge.

The BOTANICAL MAGAZINE has continued regularly to put forth its flowers every month, without regard to the severity of the season; and, since our last Report, has offered to the public a number of interesting plants, our account of which must be brief.

EUCOMIS *nana*, of the Hortus Kewensis, L'Heretier, Jacquin, and Willdenow; but Mr. Ker informs us that it has been mistakenly supposed to be the Fritillaria *nana* of Burman and Linnæus, which is the Eucomis *bifolia* (Bot. Mag. No. 840), and that it is in reality the F. *regia* of these authors, but not of the Hortus Kewensis, where Dillenius's figure is erroneously quoted as a synonym, but which belongs to the present plant; as is also the E. *regia* of L'Heretier, which belongs to *undulata* (Bot. Mag. 1083). Mr. Ker has taken great pains to settle the synonymy right, but has very properly left the names, as they have of late years been very generally applied.

IRIS *fulva*; the tawny or copper-colored Iris; a new species, introduced by Mr. Lyon, last year, from the banks of the Mississipi, where it is found indigenous, growing in the low grounds in the neighbourhood of New Orleans. This industrious collector has, at different times, brought over two very large cargoes of North-American plants; and we believe is gone again on the same pursuit, if his purpose be not interrupted by the unhappy war at present subsisting between this country and that of its younger brother, the government of the United States.

LACHENALIA *nervosa*, a new species, approaching very near to *purpureo-cærulea*, communicated by the Hon. William Herbert, from his collection at Mitcham.

VIOLA *rothomagensis*; a species that has been long known to the French botanists, as growing spontaneously near Rouen, and several other places, but does not seem to have been ever figured before.

LOBELIA *Speculum*. A pretty little annual, supposed to have been only of late discovered at the Cape of Good Hope, but which, from Dr. Sims's account, appears to have been long ago an inhabitant of the botanic garden at Amsterdam, and to have been described and figured by Commelin.

ROXBURGHIA *gloriosa*. A drawing of this beautiful climber has been before given to the public by Dr. Smith, in his exotic botany, who formed an idea of the structure of the flower, considerably different from that of Dr. Roxburgh, adopted by Willdenow, Persoon, and in the new edition of the Hortus Kewensis. Dr. Sims, while he has followed the last-mentioned authors in arranging this plant under the class Octandria, appears to us most inclined towards the opinion of Dr. Smith, who refers it to Tetrandria.

MIMULUS *luteus*. This species, the most beautiful yet known in the genus, was found by Dr. Langsdorff at Unashka, and transmitted to this country, as perfectly new, under the name of M. *Langsdorfii*; but, but, by Dr. Sims's account, it appears to have been described and figured long ago by Father Feuillée. We observe that the same species is probably recorded in a catalogue, printed in the present year at Moscow, under the name of M. *guttatus*. This plant probably has the merit of being perfectly hardy, and of easy propagation; so that it will no doubt be soon common, and prove a valuable addition to the flower-garden.

The last recorded number finishes the thirty-sixth volume of this charming work, containing in the whole fifteen hundred colored engravings of plants cultivated in the English gardens, all drawn from nature, chiefly by that excellent botanical draughtsman, Mr. Sydenham Edwards.

The size of this work is too small to do full justice to many of the subjects represented in it, and the want of dissections, displaying the parts of fructifications, lessens its value to the botanical student; but these defects are in some measure remedied by the ingenuity of the artist, who has generally contrived to place the different flowers in such attitudes as to show the most important parts, and are amply recompensed by the cheapness at which it is afforded. We may safely challenge the world to produce such a number of excellent botanical representations, so faithfully executed, at so reasonable a price. It is high time that the editors should favor the public with another general index; sixteen volumes having been published since the last.

Number 310 of the same work contains

TRITONIA *longiflora*, $\beta$, and $\gamma$, two additional varieties to the one before published, one of which had been formerly enumerated by Mr. Ker, as a distinct species, under the name of T. *tenuiflora*.

Tritonia *Rochensis*. A species nearly allied to the last mentioned, but still more ornamental, and never before figured. Communicated by Messrs. Lee and Kennedy.

IRIS *prismatica*. A new species, from the same collection, nearly allied, perhaps a variety only of I. *virginica*. Introduced by Mr. Pursh, a Russian botanist, who has travelled much in North America, and, as we are informed, is publishing a new Flora of that country, which will be much richer than that of Michaux.

NARTHECIUM *americanum*. This plant is a congener of Anthericum *ossifragum* of Linnæus, which latter species was first separated from Anthericum by our Hudson, whose name does not however occur in this account, Mr. Ker having adopted the generic character of a late writer, Wahlenberg, in his Flora Lapponica. The Narthecium of Jussieu, Michaux, and the French writers in general, is another genus, the Tofieldia of Hudson, Anthericum *calyculatum* of Linnæus. Inattention to this circumstance probably led Mr. Pursh and Mr. Ker to suppose that this plant was the Narthecium *glutinorum* of Michaux, under which name it was first given in the Botanical Magazine; but corrected in the following number.

CALOTHAMNUS *quadrifidi*. A handsome shrub of the natural order of *Myrti*, and nearly related to Melaleuca. The genus was first established by M. Labillardiere, or rather the name for Mr. Brown's

Brown's definition of the genus, given in the Hortus Kewensis, includes several species, which that of the former would not, of which this is one.

Billardiera *longiflora.* Another elegant New-Holland climber. The fruit of this species is very different both in shape and color from B. *scandens*, to which the plant is otherwise in many respects like.

Platyloaium *triangulare.* Another beautiful shrub, from the same country, and never before figured.

Aster *liratus.* A new species, now first described, still from the same country.

Pomaderris *elliptica;* the Ceanothus *discolor* of Ventenat, by whom there is a figure of it published in the Jardin de Malmaison. This being a native of Van Diemen's Island, Dr. Sims remarks, would probably, in a sheltered situation, bear the cold of our winters very well.

Bignonia *uncata.* Native of the West-India islands, and an ornamental stove climber. It was introduced by Lord Seaforth, not long since governor of St. Vincents, and a great promoter of the science of botany. Communicated from the magnificent collection of Madame the Comtesse de Vandes.

Satyrium *carneum.* Orchis *carnea* of the first edition of Aiton's Hortus Kewensis, and one of the most beautiful of the genus. Communicated by Mr. Griffin, who has a capital collection of Cape plants at South Lambeth.

Jeffersonia *diphylla;* the Podophyllum *diphyllum* of Linnæus, which name is continued to it in the last edition of the Hortus Kewensis, notwithstanding Dr. Barton and Michaux had long before separated it from that genus, between which and Sanguinaria it seems to balance; Dr. Sims thinks, that, of the two, it would unite best with the latter; but its fruit is remarkably different from both.

Iris *desertorum* of Pallas, which Mr. Ker considers as a variety only of Iris *spuria.* This is at present a very scarce plant, but recommends itself by the extreme fragrance of its flowers. Communicated from Mr. Middlemist's nursery at Shepherd's-bush.

Iris *stenogyna* of Ridouté. Mr. Ker considers this likewise as a variety of Iris *spuria.* Communicated by Mr. Donn, from the Cambridge botanical garden.

Galaxia *ovata*, var. *purpurea.* This is altogether a smaller plant than the yellow variety before figured in the Magazine; and we should be much inclined to consider it as a distinct species. In the drawings there are some remarkable differences in the foliage and in the comparative length of the tube of the corolla; but without comparing the living plants we cannot pretend to determine.

Lachenalia *racemosa;* a species now first described, nearly related to L. *pustulata.*

It is with pleasure we announce to our readers, that the fourth volume of the new edition of the Hortus Kewensis is printed, although its publication has not yet taken place, and perhaps may be deferred till the fifth shall be finished, which will complete the work.

